# Surgical Management of Complications after Dexamethasone Implant

**DOI:** 10.1155/2020/4837689

**Published:** 2020-02-10

**Authors:** Giancarlo Sborgia, Alfredo Niro, Francesco D'Oria, Alessandra Galeone, Luigi Sborgia, Francesco Boscia, Alessandra Sborgia, Giovanni Alessio

**Affiliations:** ^1^Department of Medical Science, Neuroscience and Sense Organs, Eye Clinic, University of Bari, Bari, Italy; ^2^Eye Clinic, Hospital “S. G. MOSCATI, ” ASL TA, Taranto, Italy; ^3^Department of Surgical, Microsurgical and Medical Sciences, Eye Clinic, University of Sassari, Sassari, Italy

## Abstract

**Purpose:**

To report surgical management of ocular complications occurred after dexamethasone (DEX) implant (Ozurdex®) injection.

**Methods:**

Retrospective interventional case series.

**Results:**

Different surgical procedures including viscoexpression to manage the migration of the implant into the anterior chamber, “phaco-rolling” technique for the intralenticular injection, and vitrectomy with implant removal for an acute endophthalmitis were performed. Successful management of different complications after DEX implant by using individualized surgical approaches was observed.

**Conclusions:**

Early and targeted surgical management is required in selected cases of ocular complications after DEX implant. The implant removal was needed to preserve ocular anatomy and function.

## 1. Introduction

The Ozurdex® (Allergan, Irvine, CA, USA) is a 700 mcg dexamethasone (DEX) biodegradable ocular implant, formulated in a solid polymer drug delivery system, designed to be injected into the vitreous using a 22-gauge applicator device. It is used to treat macular oedema (ME) related to diabetic retinopathy, noninfectious uveitis, and retinal vein occlusion [1]. Major complications of this steroid implant include conjunctival haemorrhage, induction or worsening of cataract, glaucoma, retinal detachment, and endophthalmitis [2]. Desegmentation of the implant, lenticular injury during implantation, and migration of the implant into the anterior chamber have been described as rare but known complications [3–7]. We reported a case series of rare ocular complications related to DEX implant, successfully managed by individualized surgical approaches.

## 2. Report of Cases

This retrospective interventional case series was conducted in the Eye Clinic of University of Bari, Bari, Italy. A total of 1066 cases underwent DEX implant between October 2015 and October 2018 were reviewed. Implant was performed to treat ME secondary to Irvine-Gass syndrome, central or branch retinal vein occlusion, noninfectious uveitis, and diabetic retinopathy. Mayor ocular complications occurred in only four eyes (4/1066; 0.37%). We identified two patients who experienced the migration of the implant into the anterior chamber, one patient with intralenticular implant and one patient with acute endophthalmitis. Patient's age ranged from 60 to 81 years. All these patients underwent a comprehensive ophthalmic examination that included best-corrected visual acuity (BCVA) measurement, slit-lamp examination, intraocular pressure (IOP) measurement, spectral domain optical coherence tomography (SD-OCT), and fundus examination. Ocular ultrasonography was performed when ocular fundus cannot be examined by ophthalmoscopy. We reported targeted surgical management of three patients that experienced ocular complications after DEX implant. The study adhered to the Tenets of the Declaration of Helsinki.

### 2.1. Case 1

**Figure 1:**
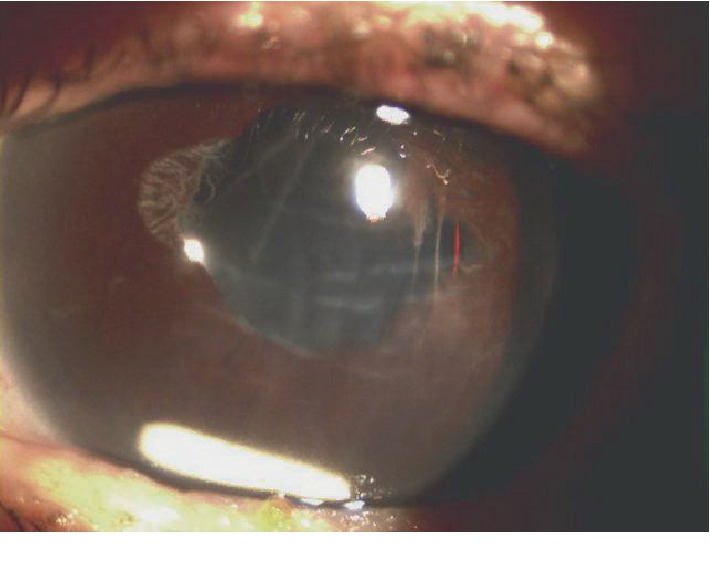
Slit-lamp photograph showing dexamethasone implant migration into the anterior chamber, corneal oedema, and Descemet's membrane folds.

An 81-year-old Caucasian woman underwent a complicated cataract surgery with posterior capsule rupture and sulcus intraocular lens (IOL) implantation. Three months after surgery, a central retinal vein occlusion with ME occurred in the same eye and five consecutive intravitreal injection of anti-Vascular Endothelial Growth Factor (VEGF) were delivered monthly. Due to a poor therapeutic response, she was planned to perform an intravitreal DEX implant. Three weeks following the implant, she complained decreased vision. Visual acuity was limited to 20/200, and IOP was 25 mmHg. We found implant migration into the anterior chamber, and corneal oedema and Descemet's membrane folds were observed (Figure 1). After the informed consent was obtained, the patient underwent surgery by the “viscoexpression” technique, as described by Rahimy et al. [8]. Under topical anesthesia, a clear corneal incision of 2.75 mm opposite from the implant localization was performed. Intracameral injection of acetylcholine chloride was performed to constrict the pupil and avoid the migration of the implant back into the posterior chamber. A dispersive/adhesive ocular viscoelastic device (OVD) was inserted into the anterior chamber to avoid damaging adjacent structures and to facilitate the extrusion of the implant through the paracentesis. Viscoelastic was injected initially through the tunnel in the space between the implant and corneal endothelium. The continuous injection behind the implant maintains the chamber under pressure and pushes the implant toward the incision. Simultaneously, a pressure was posteriorly directed over the corneal incision by the cannula, opening the tunnel and engaging the implant into the tunnel. The OVD was then removed from the anterior chamber using irrigation with balanced salt solution. After two weeks, BCVA was 20/63. Slit-lamp examination revealed the resolution of Descemet's folds and corneal oedema, without any other signs of inflammation in the anterior chamber. Patient required to switch to intravitreal anti-VEGF to treat ME.

### 2.2. Case 2

A 67-year-old diabetic Caucasian man underwent DEX implant to treat diabetic ME, as the first treatment. At 1-week follow-up, we observed DEX rod impeded into the lens. BCVA was 20/200. IOP was 30 mmHg unresponsive to topical therapy. After the informed consent was obtained, phacoemulsification was performed. The nucleus and the implant were removed using a variation of “phaco-rolling” technique firstly described by Güell et al. [9]. A temporal clear corneal incision of 2.75 mm was performed. The anterior chamber was filled with a viscosurgical device. A 6.0 mm continuous curvilinear capsulorhexis was made, after which hydrodissection and hydrodelamination were performed. A complete rotation of the lens within the capsular bag was performed. A 15-degree phaco tip was placed on the lens beside the capsulorhexis edge and in contact with the nucleus-epinucleus surface, so phacoemulsification was started. Then, we modified the inclination of the tip to 45 degree, directing it toward the lens center. High aspiration was used to keep the probe tip occluded. We used a peristaltic pump for phaco rolling. The lens began to rotate, and phacoemulsification was performed from the periphery toward the center so the nucleus and the implant in the capsular bag were emulsified. No posterior capsular defect was observed intraoperatively. A single-piece intraocular lens (IOL) was placed into the capsular bag, followed by an intravitreal DEX implant ([Supplementary-material supplementary-material-1]). At one-month follow-up, ME was reduced, BCVA significantly improved up to 20/32, and IOP was 18 mmHg.

### 2.3. Case 3

**Figure 2:**
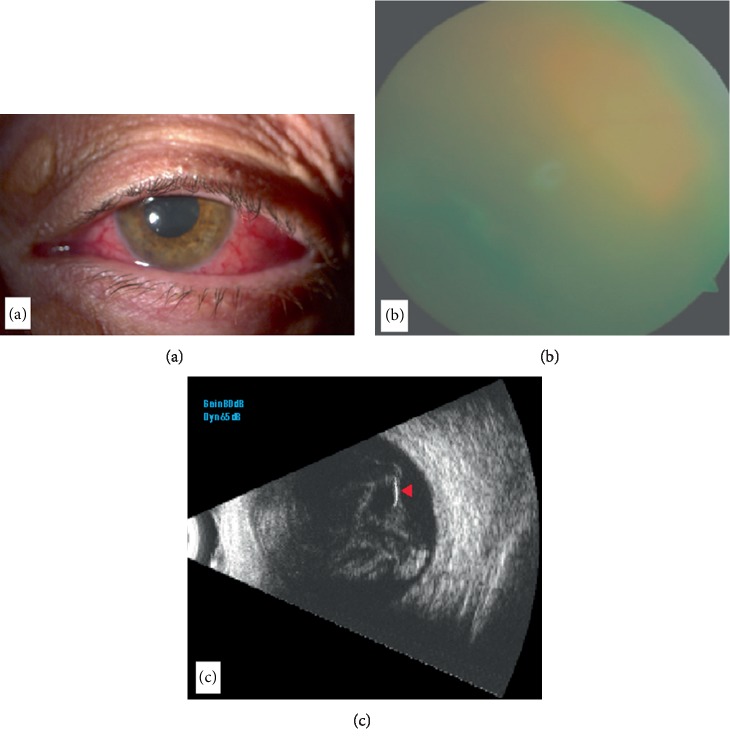
(a) Slit-lamp photograph showing anterior segment reaction. (b) Vitreous cells and severe vitreous haze hinder funduscopy. (c, d) Ocular ultrasonography B-scan reveals the device as a hyperreflective area (arrowhead) surrounded by dense hyperreflective opacities as dots and membranes in the vitreous cavity.

A 60-year-old male patient underwent combined phacoemulsification with in-the-bag hydrophilic acrylic IOL implantation followed by DEX implant for cataract and diabetic ME in the left eye. Five days after surgery, the patient complained pain, redness, and vision loss in the treated eye. Visual acuity was limited to light perception. Slit-lamp examination revealed ciliary injection and 2+ anterior chamber cells. The retinal reflex was poor. B-scan ultrasonography showed high iperecogenous echi with mobile membranes in the vitreous chamber (Figure 2). The diagnosis of acute endophthalmitis was made. After the informed consent was obtained, surgery was performed. Aqueous sample was collected for microbiological evaluation. Then, we performed a standard 3-port 25 Gauge pars plana vitrectomy. Vitreous was aspirated before starting the infusion to get an undiluted sample. The machine was set with an initial aspiration of 0 mmHg moving linearly to 650 mmHg when the foot pedal was fully depressed. A “core” vitrectomy was performed followed by induction of posterior vitreous detachment. The implant was settled peripherally in the vitreous chamber. Vitreous adhesions were removed from the implant with low aspiration and high cut rate. Perfluorocarbon liquid was injected in order to protect the macula. The implant was easily engaged and anteriorized lowering the cut at 0 cut/min and aspiration at 550 mmHg. The IOL was subluxated using a hook introduced through a 1.4 mm superior sclero-lumbar access and the implant was moved to anterior chamber through a posterior capsule tear observed intraoperatively. The sclero-lumbar access was enlarged to 2.4 mm, and the implant was extracted by applying a gentle pressure on the edge of the tunnel. After an extensive vitrectomy, the patient received an intravitreal injection of vancomycin (1 mg/0.1 mL) and amikacin (0.4 mg/0.1 mL) followed by silicone oil injection ([Supplementary-material supplementary-material-1]). The patient was placed on topical Tetracycline 1% six times a day, Dexamethasone 0.1% four times a day, and intravenous Imipenem and Cilastatina Hikma 500 mg four times a day. Vitreous cultures were negative, but aqueous tap yielded Kocuria Rhizophila and Staphylococcus Epidermidis that were sensible to all the antibiotics tested at the antibiogram. After 5 days, ocular inflammation significantly decreased and the patient was discharged with topical antibiotic and a tapering regimen of steroid eye drops. At two-month follow-up, no signs of infections were detected and BCVA improved to 20/63.

## 3. Discussion

In a retrospective review of 924 DEX implantations performed, Kang et al. reported four cases of anterior chamber migration in the presence of a compromised posterior capsule, [10] as we observed. In another previous report, corneal oedema was observed when anterior migration occurred over 3 weeks after implant. Authors suggested that corneal oedema was due to mechanical trauma of the implant on corneal endothelial layer rather than chemical toxicity. So the earlier removal of the implant was performed to reduce the risk of irreversible corneal endothelial damage [6]. In our patients who experienced anterior chamber migration, ocular symptoms occurred from 3 to 4 weeks after injection when corneal oedema was observed.

Various procedures to remove the implant from anterior chamber such as the aspiration of the disintegrated implant, the use of forceps, viscoelastic material, or IOL injector when the implant is still stiff were reported [6–8]. We performed viscoexpression using OVD without any contact with the implant. This approach ensures that no vehicle particles are left behind after the intervention [8]. We did not perform a repositioning of the implant due to the severe corneal oedema and the risk of migration recurrence. However, the repositioning of the implant into the vitreous cavity may be firstly attempted in the absence of severe corneal decompensation [11]. The accidental injection into the lens is an unexpected complication that occurs rarely. Coca-Rabinot et al. reported a case series of two patients with accidental injection into the lens associated with raised IOP responsive to the topical therapy. Only when cataract occurred after several weeks, authors performed cataract surgery [6]. In our case, we detected an increased IOP not responsive to topical therapy, so we decided to perform phacoemulsification. We decided to perform the “phaco-rolling” technique that allows to reduce phaco time and phaco energy compared to divide-and-conquer technique, as our standard technique, maintaining a constant irrigation/aspiration [9]. To our knowledge, for the first time, “phaco-rolling” technique was used to remove DEX implant embedded into the lens. We tried to reduce the risk of damage to the corneal endothelium and posterior capsule, accidental fragmentation and dispersion of the implant during lens sculpture and removal, and postoperative complications as cystoid macular oedema. Moreover, the implant localized into the lens does not work because of the altered location [5]. The incidence of endophthalmitis after intravitreal implant is low, ranging from 0.019% to 1.4% [12], and no cases of endophthalmitis had been reported in the GENEVA study, one of the main study trials about DEX implant [13]. A study by VanderBeek et al. concluded that the incidence of endophthalmitis was higher with intravitreal injection of steroids than anti-VEGF. They stated that this could be due to the larger wound outlined by the 22-gauge needle used in steroid injection which could allow an easy bacterial penetration into the vitreous. Furthermore, since steroids have an immunosuppressive action, they might contribute to the growth of infectious agents [14]. In our last case, despite all prophylactic procedures were performed, five days after surgery, endophthalmitis occurred. The diabetic status of patient is a known risk factor for developing infections due to a deficiency of the immune system related to diabetes [15]. Moreover, the rupture of the posterior capsule, that we found, is associated with an increase of the risk of endophthalmitis [16]. The use of vitrectomy is recommended for severe and sight-threatening endophthalmitis, and in particular, early vitrectomy has been associated with better prognosis [17]. We decided to remove the implant moving it from the posterior chamber to the anterior chamber through a rupture of the posterior capsule without damaging the IOL. So we avoided to loss some fragments of the implant as a vehicle of infection if we had used the vitrectome. Oil tamponade limiting the movements of aqueous humor and, as an inert material, the penetration of microorganisms can favor better outcomes [18]. The opportunity of removing instead of repositioning the implant should be considered in case of corneal oedema endophthalmitis or when implant does not work. We performed different surgical maneuvers, of which two were never used before in the management of DEX implant complications to avoid implant fragmentation and damages of intraocular structures.

## Supplementary Materials

Supplementary 1 Video 1.mp4: phaco-rolling technique for intralenticular implant.

Supplementary 2 Video 2.mp4: vitrectomy for endophthalmitis after DEX implant.
